# The CWI Pathway: Regulation of the Transcriptional Adaptive Response to Cell Wall Stress in Yeast

**DOI:** 10.3390/jof4010001

**Published:** 2017-12-21

**Authors:** Ana Belén Sanz, Raúl García, José M. Rodríguez-Peña, Javier Arroyo

**Affiliations:** Departamento de Microbiología II, Facultad de Farmacia, Universidad Complutense de Madrid, Instituto Ramón y Cajal de Investigaciones Sanitarias (IRYCIS), 28040 Madrid, Spain; absanzsa@ucm.es (A.B.S.); rgarcias@ucm.es (R.G.); josemanu@ucm.es (J.M.R.-P.)

**Keywords:** cell wall integrity, mitogen-activated protein kinase (MAPK), signal transduction, transcription, gene expression, antifungal

## Abstract

Fungi are surrounded by an essential structure, the cell wall, which not only confers cell shape but also protects cells from environmental stress. As a consequence, yeast cells growing under cell wall damage conditions elicit rescue mechanisms to provide maintenance of cellular integrity and fungal survival. Through transcriptional reprogramming, yeast modulate the expression of genes important for cell wall biogenesis and remodeling, metabolism and energy generation, morphogenesis, signal transduction and stress. The yeast cell wall integrity (CWI) pathway, which is very well conserved in other fungi, is the key pathway for the regulation of this adaptive response. In this review, we summarize the current knowledge of the yeast transcriptional program elicited to counterbalance cell wall stress situations, the role of the CWI pathway in the regulation of this program and the importance of the transcriptional input received by other pathways. Modulation of this adaptive response through the CWI pathway by positive and negative transcriptional feedbacks is also discussed. Since all these regulatory mechanisms are well conserved in pathogenic fungi, improving our knowledge about them will have an impact in the developing of new antifungal therapies.

## 1. Cell Wall Organization and Structure in *S. cerevisiae*

Yeast cell integrity depends on the cell wall, an essential structure necessary not only for maintaining morphology but also for protecting cells against environmental stress conditions [1,2]. The cell wall is a macromolecular complex mainly composed of β-1,3-glucan, β-1,6-glucan, chitin and mannoproteins. Chitin, although a minor component, is essential for cell survival. β-1,3-glucan is the most abundant component of the yeast cell wall and serves as a backbone to which the other cell wall components are linked. The reducing ends of β-1,6-glucan and chitin are attached to the non-reducing end of β-1,3-glucan chains by an uncharacterized link and a β-1,4-linkage, respectively [3,4,5]. β-1,6-glucan is also attached to the chitin through β-1,3-linked oligogluco-residues that branch off the glucan. There is also a fraction of free chitin. Cell wall mannoproteins, including those involved in adhesion, cell wall remodeling, structural proteins and somatic antigens [6,7,8,9] can be linked directly to the β-1,3-glucan via alkali-labile bonds [9,10] and indirectly via β-1,6-glucan through a glycosylphosphatidylinositol (GPI) anchor [9,11]. The structure of the fungal cell wall is very well conserved among fungi despite several differences in cell wall composition exist [12]. Among fungal glucans, diversities in configuration, position of glyosidic bonds and branching have been reported [13]. β-1,3-glucan is the major cell wall component and is present in all the fungal species analyzed, whereas other polysaccharides including β-1,3/β-1,4-glucan, β-1,6-glucan or α-1,3-glucan are found in some but not all fungal cell walls [14]. For example, β-1,6-glucan is present in *Saccharomyces cerevisiae* and *Candida albicans* [15,16], although it is absent in *Aspergillus fumigatus* [17]. In contrast, galactomannan and β-1,3/1,4-glucan are present in *Aspergillus* but not in yeast [18]. α-1,3-glucan plays an important role in the organization of the cell wall of *Schizosaccharomyces pombe* and many human pathogens but is absent from the *Candida* and *Saccharomyces* cell walls [12,14].

The essentiality of the cell wall for fungal growth viability and virulence together with the fact that most of the fungal cell wall components and the corresponding enzymatic activities necessary for cell wall biogenesis and remodeling are not present in mammals points this structure as an ideal target for therapeutic intervention against fungal infections [12,19]. Indeed, compounds belonging to the antifungal family of echinocandins, such as caspofungin, anidulafungin and micafungin, are able to selectively inhibit the synthesis of β-1,3-glucan in the cell wall [20] and are currently in clinical use [21]. However, the efficacy of these drugs can be offset by the induction of fungal cell wall compensatory mechanisms [22,23,24,25].

## 2. Yeast Adaptive Response to Cell Wall Stress: The CWI Pathway

The cell wall is a very dynamic structure whose properties vary constantly to be adapted to different growth conditions. The cell wall composition changes with the nature of carbon source, nutrient or oxygen availability, temperature or external pH [26,27]. Regulation of cell wall synthesis is part of cellular growth programs. The cell must remodel this structure to adapt cell growth during vegetative proliferation, mating pheromone-induced morphogenesis and bud formation, and hence cell division. In accordance, the transcription of many cell wall genes varies during the cell cycle and particularly at G1 for isotropic cell wall synthesis [15]. Cell wall remodeling includes breakage of preformed cross-links and the incorporation of new synthesized polysaccharides into the preexisting cell wall core by elongation, branching and cross-linking through transglycosylation enzymatic activities.

In particular, the cell wall structure needs to be remodeled under those stress conditions with a direct impact on cell wall integrity [28]. Therefore, treatments with cell wall perturbing agents that directly induce cell wall stress by interfering cell wall construction or mutational loss of proteins necessary for cell wall biogenesis elicit a rescue mechanism known as “compensatory salvage response” that provides compensatory synthesis of cell wall material and changes in the cross-linking between cell wall polymers necessary for maintenance of cellular integrity and fungal survival [1,29]. Among those chemical agents that interfere with cell wall biogenesis inducing cell wall stress in yeast are: Congo red and Calcofluor white, both of them binding to chitin and interfering with proper cell wall construction [30,31]; zymolyase which alters the cell wall through its β-1,3-glucanase, protease and chitinase activities [32] or echinocandins which inhibits β-1,3-glucan synthase [20]. Cell wall remodeling is also induced in response to a variety of environmental stresses that indirectly affect cell wall integrity and therefore activate the stress adaptive rescue responses, including heat, ethanol, osmotic or oxidative stresses [26,33].

One of the best characterized mechanisms included in the yeast compensatory response consists of variations in cell wall polysaccharide contents, the most remarkable being the increase in the chitin fraction [29,34]. Cells with mutations affecting the synthesis (*fks1*Δ) or elongation (*gas1*Δ) of β-1,3-glucan, as well as synthesis of β-1,6-glucan *(kre6*Δ), mannan (*mnn9*Δ), *O*-linked glycans and GPI anchors respond by depositing additional chitin in their lateral cell walls in compensation for compromised cell integrity [26,28,35,36,37,38]. In addition to an induction of chitin content, heat shock and caspofungin treatments also causes an increase in the β-1,6-glucan polysaccharide fraction and a partial reduction of β-1,3-glucan [26]. Compensatory mechanisms also include: an increment in the amount of several cell wall proteins [39,40], transient re-distribution of β-1,3-glucan synthase complex through the cell [41,42] and changes in the cross-linking between cell wall polymers [43]. In this sense, the proportion of chitin bound to β-1,6-glucan raises significantly under heat stress conditions whereas the chitin-β-1,3-glucan fraction decreases [6]. Moreover, increased resistance to zymolyase has been found under heat, ethanol, osmotic or oxidative stress conditions [26,33], suggesting an active cell wall remodeling.

The aim of all these changes triggered in the compensatory response must be to reinforce the cell wall by rebuilding cell wall blocks making them less flexible. In fact, overexpression of cell wall remodeling enzymes reduces fungal cell wall elasticity and increase osmotic stress resistance [44]. As discussed in more detail below, many of these changes are the result of a transcriptional reprogramming elicited by the yeast as a consequence of cell wall stress, that include among others, the induction of a large group of genes related to cell wall biogenesis [30,45].

**Figure 1:**
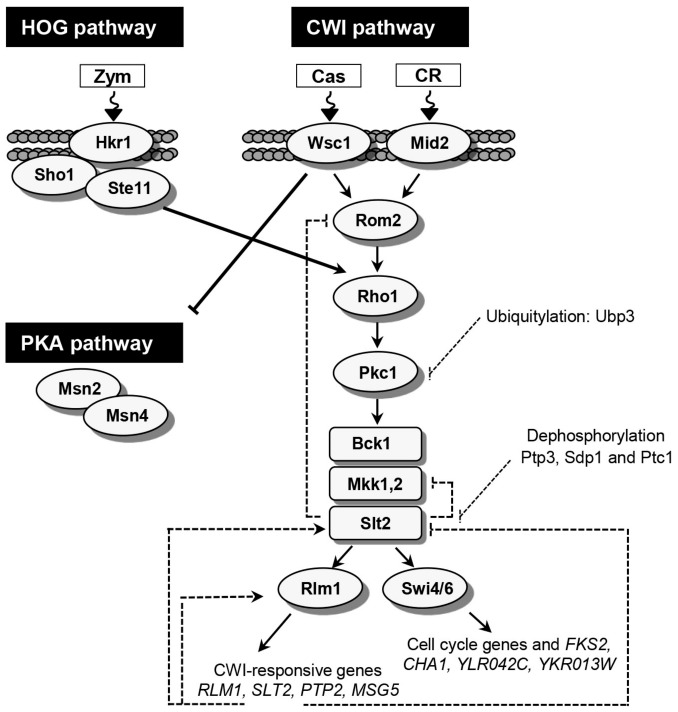
The cell wall integrity (CWI) signaling pathway. Cell wall damage is sensed (wave arrows) at the plasma membrane through cell-surface proteins that stimulate nucleotide exchange on Rho1 and activation of Pkc1. The main role of activated Pkc1 is to trigger the MAPK module (Bck1, Mkk1/Mkk2 and Slt2). Once phosphorylated, the MAPK Slt2 activates two different transcription factors, SBF (Swi4/Swi6) and Rlm1. Cell wall damage caused by Congo red (CR) is sensed mainly through Mid2 to activate the CWI pathway; Zymolyase (Zym) mediated cell wall stress requires Hkr1 sensor and elements of the Sho1 branch of the high-osmolarity glycerol (HOG) pathway to activate Rho1; Caspofungin (Cas) is sensed through Wsc1 leading to the activation of the CWI pathway and parallel inhibition of protein kinase A (PKA) signaling (solid lines) Modulatory mechanisms of CWI signaling are represented with broken lines. Rlm1 elicits transcriptional positive feedback loops on the expression of *RLM1* and *SLT2*. In contrast, attenuation of the CWI pathway requires negative retrophosphorylation feedback loops mediated by Slt2 on Rom2 and Mkk1/2 and the Rlm1-dependent transcriptional induction of the Slt2 phosphatases Ptp2 and Msg5. In addition, other Slt2 phosphatases like Ptp3, Sdp1 and Ptc1 and ubiquitination of Pkc1 by Ubp3 also contribute to attenuate the induction of CWI pathway. Arrows and T symbols represent activation (positive) and inhibitory (negative) events, respectively.

Several signaling cascades has been involved in the regulation of cell wall stress responses, but the yeast cell wall integrity (CWI) pathway, governed by the mitogen-activated protein kinase (MAPK) Slt2, is key for this purpose [1]. This pathway is very well conserved in the fungal kingdom [46]. It consists of a group of several plasma membrane sensors, Wsc1-3, Mid2 and Mtl1 that interact with the guanine nucleotide exchange factor (GEF) Rom2 activating the small GTPase Rho1, which then activates the protein kinase C (Pkc1). Pkc1 triggers a conserved MAPK module phosphorylating the MAPKKK Bck1 which activates a redundant pair of MAPKKs Mkk1/Mkk2 which finally phosphorylates and activates the MAPK Slt2 (also known as Mpk1). Slt2 acts on two transcription factors: Rlm1 [47] and SBF (Swi4/6) [48]. SBF is mainly involved in the regulation of genes during G1/S transition whereas Rlm1 is responsible for the transcriptional activation of most of those genes induced in response to cell wall stress (Figure 1).

CWI signaling can be monitored quantifying the phosphorylation of Slt2. Those stress conditions mentioned above with a direct impact on cell wall integrity, including treatments with chemical agents or mutations affecting cell wall biogenesis induce CWI signaling. The MAPK is also activated under a variety of other stress conditions that indirectly affects cell wall, including heat stress, hypo-osmotic shock, hyper-osmotic shock, ER stress, oxidative stress, high and low pH, DNA–damaging agents, etc. [1]. In some of these cases the mechanism by which Slt2 is activated is not completely understood.

## 3. The Transcriptional Program to Cell Wall Stress: Regulatory Mechanisms

DNA microarray and RNA-sequencing technologies have been powerful tools for the characterization of genome-wide transcriptional profiles, being an important step for the understanding of transcriptional stress responses. The first study focused on the characterization of the global transcriptional response under activation of the CWI pathway used a hyperactive allele of Mkk1 (*MKK1^S386P^*). This study revealed that constitutive activation of the CWI pathway, through the activation of the transcription factor Rlm1, induces the expression of approximately 25 genes, most of them related to cell wall biogenesis [40]. Hierarchical clustering of differentially expressed genes in different cell wall related mutants (*gas1*Δ, *mnn9*Δ, *fks1*Δ, *kre6*Δ and *knr4*Δ) with a constitutive Slt2 hyperactivation identified a stereotypical transcription response to cell wall damage, containing a set of almost 80 genes [45]. Other studies covered the analysis of the yeast response to transient cell wall stress by characterization of the transcriptional program to treatments with cell wall perturbing agents that interfe with cell wall biogenesis through different mechanisms of action like Congo red [30], Calcofluor white [49], zymolyase [50], caspofungin [51,52,53,54] or compounds like OGT2468 that inhibits *O*-mannosylation and affects cell wall mannoproteins [38]. Comparison of all these responses, which includes induction of approximately 100–200 genes, reveals the existence of both specific and common transcriptional profiles. Indeed, the cluster of genes induced in almost all conditions could be considered as a “transcriptional fingerprint” for cell wall stress [55]. This signature mainly comprises the induction of genes related to cell wall biogenesis and remodeling (*SED1*, *CWP1*, *GFA1*, *YLR194C*, *PST1*, *PIR3*, *CRH1*, *HSP150* and *CCW14*), metabolism and energy generation (*YHR209W* and *YPL088W*), morphogenesis (*DFG5*), signal transduction (*SLT2*, *MLP1, RLM1*) and stress (*HSP12* and *YIL117C*) [55].

Although the immediate role of gene expression in rapid adaptation is still being discussed, it is accepted that gene expression is important for long-term adaptation to stress and for protection against future stress [56,57]. In the case of the cell wall stress response, the transcriptional induction of cell wall related genes clearly reflects the necessity of cell wall remodeling. As detailed above, the induction of chitin synthesis as a consequence of cell wall stress is one of the main compensatory fungal responses. In agreement, genes encoding enzymes of the chitin synthesis pathway like *GFA1*, which encodes glutamine-fructose-6-phosphate amidotransferase, the first committed enzyme of this pathway [58] and *CHS3*, encoding the catalytic subunit of the chitin synthase III [59], are overexpressed in almost all the conditions referenced above. *CRH1*, which is also overexpressed in all cell wall stress conditions tested so far [60], encodes for a transglycosylase involved in the formation of chitin-glucan cross-links [6,61]. Thus, strengthening of cell wall under stress by overproduction of chitin also requires the covalent association of the chitin synthesized to the glucan network through the action of this activity which is induced at the transcriptional level [60]. In fact, a temperature shift from 30 °C to 38 °C increases the fraction of chitin attached to β-1,6-glucan as a consequence of *CRH1* overexpression and relocalization of Crh1 and the homologous Crh2 to the lateral cell wall [6]. The overexpression of non-enzymatic structural cell wall proteins like Sed1, Cwp1, Pst1, or Ccw14, as consequence of the compensatory response, clearly reflects their collective role in maintaining cell wall stability [15,62].

Among the signaling-related genes overexpressed during most of the cell wall stress conditions there are: *SLT2*, which encodes for the CWI MAPK, *MLP1*, which encodes for a pseudokinase paralog to Slt2, and *RLM1*. Rlm1 is the transcription factor responsible for the expression of the majority of the genes induced under cell wall stress through the CWI pathway as deduced from global profiling of strains deleted in *RLM1* when compared to a wild-type strain [30,63]. The transcriptional activity of Rlm1 is regulated by Slt2-mediated phosphorylation of residues Ser427 and Thr435 [64]. Binding of phosphorylated Rlm1 to the promoters of Rlm1-dependent genes and assembly of the transcription initiation machinery require complete nucleosome remodeling at these promoters that is achieved through the cooperation between the ATP-dependent chromatin remodeling complex SWI/SNF and SAGA complex [63,65].

In addition to transcription of G1/S related genes, SBF (Swi4/Swi6) regulates the expression of a small group of CWI genes, including *FKS2*, *CHA1*, *YLR042C* and *YKR013W,* through a non-catalytic mechanism [66,67,68]. This mechanism requires the activation of Slt2 (but not its catalytic activity) and Mlp1 to interact with Swi4 and direct both to SBF-binding sites independently of Swi6. Slt2 and Mlp1 travels along the coding region associated with the Pol II and Paf1C to avoid premature termination by the Sen1-Nrd1-Nab3 complex [66].

Genome-wide transcriptional analysis of yeast mutant strains deleted in different components of CWI and other signaling pathways, like the high osmolarity glycerol (HOG) and PKA pathways, allowed the identification of critical regulatory elements for the cell wall transcriptional responses. In this way, subtle differences in the regulatory mechanisms involved depending on the type of stress were found. The Congo red-mediated damage is sensed through Mid2 and elicits a transcriptional response through the CWI pathway that depends almost completely on the MAPK Slt2 and the transcription factor Rlm1 [30,53,63] (Figure 1). Cell wall damage caused by zymolyase also induces a main response related to adaptation to cell wall integrity defects regulated by Slt2 and Rlm1. However, this response requires previous activation of elements of the HOG pathway. The yeast adaptive program to hyperosmotic stress that includes temporary arrest of cell-cycle progression, adjustment of transcription and translation patterns, and the synthesis and retention of glycerol, is governed by the HOG pathway [69]. Under zymolyase-mediated cell wall damage, phosphorylation of Slt2 depends on components of the Sho1 branch of the HOG pathway, and it requires essential components of the CWI pathway as Mkk1/Mkk2, Bck1 and Pkc1 but not any of the membrane sensors and guanine nucleotide exchange factors belonging to this pathway. In fact, zymolyase damage is sensed through Hkr1 [32], the sensor of the Sho1 branch of the HOG pathway. Thus, in agreement with the model of sequential activation of the HOG and CWI pathways, activation of Slt2 by a previous activation of the elements of the Sho1 branch of the HOG pathway is responsible, through Rlm1, for the majority of cell wall transcriptional adaptive response to zymolyase [50,53,70] (Figure 1).

Cell wall damage caused by the echinocandin caspofungin, an inhibitor of β-1,3-glucan synthase, is sensed through Wsc1 [51,52] and the transcriptional response under this condition is completely dependent on this sensor [53,54]. The global transcriptional response elicited by inhibition of the β-1,3-glucan synthesis includes a set of genes dependent on the MAPK Slt2 and the transcription factor Rlm1 and a broad group of genes regulated independently of Slt2. Cell wall alterations derived from this stress induce, through Wsc1, the activation of the CWI pathway and parallel inhibition of PKA signaling [51,52,53,54] (Figure 1). Activation of Slt2 triggers the corresponding CWI transcriptional response. Negative regulation of PKA signaling promotes nuclear localization of Msn2 and cellular glycogen accumulation, a decrease of intracellular cAMP and active GTPase Ras2 levels, together with a reduction in the phosphorylation of known substrates of PKA, all these effects relying on the Wsc1 sensor [54]. In consequence, the transcriptional induction of Slt2-independent transcripts in response to caspofungin correspond to genes that exhibit upregulated expression in conditions where the cAMP/PKA pathway is deactivated.

All these studies reflect the importance of the CWI pathway and the input received by other pathways to overcome cell wall stress situations, showing the complexity of the signal transduction machinery responsible for this regulation.

## 4. Modulation of the CWI Pathway through the Transcriptional Response

The timing of the yeast cell wall stress adaptive response mediated by the CWI pathway and the development of the corresponding transcriptional response is very different to that of other MAPK signaling pathways like the HOG pathway or the mating signaling pathway. In contrast to the rapid and transient activation of Hog1 following osmostress [69], CWI signaling is activated persistently at elevated temperatures or treatment with cell wall perturbing agents. Thus, cell wall damage caused by direct cell wall perturbing agents Calcofluor white, Congo red and zymolyase elicits a slow and sustained response over time. Under these cell wall stress conditions, the MAPK Slt2 is phosphorylated within 15 min upon stress and this activation reaches a maximum value after 1 to 2 h. After treatment, the phosphorylation of the MAPK decreases but it can still be detected even after 6 h [30,39,70]. Gene expression pattern correlates with Slt2 activation. Thus, after one hour of cell wall stress there are some genes whose expression is induced, being a maximum response reached after 2–3 h [30]. Moreover, the kinetic response indicates the existence of a transient response for one group of genes which includes those related to signal transduction, and a more sustained response including those involved in cell wall construction and remodeling [30].

Triggering sustained or transient cellular responses through MAPK signaling pathways requires precise regulatory mechanisms that define different dynamics which determine different outputs [71]. An important way of regulating these pathways is at the level of MAPK phosphorylation by protein phosphatases. In this context, Slt2 activity can be negatively regulated by dephosphorylation through the activity of different phosphatases such as Ptp2 and Ptp3 [72], Ptc1 [73,74] and the dual-specificity protein phosphatases Msg5 [75] and Sdp1, which specifically interacts with and inactivates Slt2 [76]. Interestingly, Ptp2 and Msg5 are transcriptionally induced in several cell wall stress conditions in a Slt2-dependent manner, indicating a transcriptional negative feedback at this level [30,50,72,77] (Figure 1). Additionally, other negative feedback events contribute to attenuate CWI pathway responses (Figure 1). Slt2 exerts a negative feedback by downregulation of the guanine exchange factor activity of Rom2 through a retrophosphorylation mechanism [78]. Retrophosphorylation of the MAPKKs Mkk1 and Mkk2 by Slt2 has also been reported [79]. Moreover, ubiquitination of Pkc1 by Ubp3 also contribute to attenuate the induction of the CWI pathway [80].

In contrast to negative autoregulation, which speed up transcriptional gene circuits [81], positive autoregulation that occurs when a transcription factor enhance its own production rate amplifies gene expression and slow down the kinetics of gene expression [82]. As described above, expression of *RLM1* and *SLT2* is induced by cell wall stress in an Rlm1-dependent manner [30,40,63,83]. Upon cell wall stress, Rlm1 is recruited to the promoters of *RLM1* and *SLT2* exerting positive-feedback mechanisms on the expression of both genes. Abrogation of the autoregulatory feedback mechanism on *RLM1* by site-directed mutagenesis of the Rlm1-binding box in the upstream regulatory sequence of this gene severely affects the transcriptional response elicited by activation of the CWI pathway [84]. Blockade of this positive feedback mechanism mediated by Rlm1 results in the lack of *RLM1* induction and Rlm1 protein overexpression, as well as an important reduction of its transcriptional target *SLT2*, resulting in a decrease of 60–70% in the expression levels of CWI-dependent genes. Abrogation of the transcriptional feedback exerted by Rlm1 on *SLT2*, affects overexpression of *SLT2* but not *RLM1*. This mechanism has less impact on the CWI output response than the Rlm1 autoregulatory feedback but partially contribute to regulate gene expression levels, particularly under long-term stress [84].

Therefore, the MAPK Slt2 regulates Rlm1 activation by phosphorylation and this activation is required for a proper CWI transcriptional response. However, phosphorylation of the MAPK and Rlm1 is not sufficient for a full functional CWI transcriptional response. Concurrent *SLT2* and *RLM1*-mediated positive feedback mechanisms are also required. Sustained patterns of gene expression are mainly achieved by these positive autoregulatory circuits based on continued activation of Rlm1.

## 5. Perspectives

Antifungal resistance represents a major challenge for treating invasive fungal infections due to the limited arsenal of available antifungal agents [85]. The discovery of novel antifungal drugs relies on the discovery of unexplored targets. Current antifungals include the family of echinocandins which targets the cell wall as non-competitive inhibitors of the β-1,3-glucan synthase. Inhibitors of the chitin synthesis, although very active in vitro, are less effective in vivo and therefore are not in clinical use [86].

Yeast cell wall adaptive responses may lead to a decrease in the effectiveness of antifungal treatments targeting the cell wall. These responses are very well conserved in other pathogenic fungi [22,23]. For example, the induction of chitin synthesis as a consequence of the cell wall stress caused by caspofungin also occurs in *C. albicans* [22] and *A. fumigatus* [23,24,87,88] and can compromise the clinical treatment success [25,89,90]. Therefore, understanding cellular mechanisms of cell wall remodeling upon cell wall stress, including the cross-linking enzymes and regulatory circuits involved in fungal cell wall stress adaptation could allow the discovery of new cell wall targets and potential antifungal drugs [91,92] or potentiate currently used cell wall interfering drugs. In this sense, combination therapies that synergistically target β-1,3-glucan synthesis and other activities regulated through the compensatory response and the CWI pathway, like those executed by the Crh transglycosylase proteins which are responsible for the cross-linking between chitin and glucan [60,61], can be envisioned as an effective strategy for the development of future successful antifungal therapies. Additionally, the recently described novel connection between the CWI and the PKA pathways to regulate the cell wall stress mediated by caspofungin also points to possible combinational therapies interfering PKA regulation and cell wall integrity [54].

In addition to the clinical interest of these studies, they could also be relevant for biotechnological purposes derived from the interest in the optimization of fermentation processes [27,93] or production of cell wall polysaccharides (i.e., glucans, mannoproteins, chitin) for functional foods and pharmaceutical and cosmetic purposes [94].
